# SERPINB3 protects from oxidative damage by chemotherapeutics through inhibition of mitochondrial respiratory complex I

**DOI:** 10.18632/oncotarget.1411

**Published:** 2013-12-24

**Authors:** Francesco Ciscato, Marco Sciacovelli, Gianmarco Villano, Cristian Turato, Paolo Bernardi, Andrea Rasola, Patrizia Pontisso

**Affiliations:** ^1^ CNR Institute of Neuroscience and Department of Biomedical Sciences, University of Padova, Padova, Italy; ^2^ Department of Medicine, University of Padova, Padova, Italy; ^3^ present address: Medical Research Council Cancer Unit, Hutchison/MRC Research Centre, Hills Road, Cambridge, United Kingdom

**Keywords:** SERPINB3, chemotherapeutics, mitochondria, respiratory complexes, reactive oxygen species, cell death

## Abstract

SERPINB3 (SB3) is a serine protease inhibitor overexpressed in several malignancies of epithelial origin, including primary liver cancer, where it inhibits apoptosis through poorly defined mechanisms. In the present study we analyze the effect of SB3 on hepatoma cell death elicited by a panel of chemotherapeutic agents. We report that SB3 shields cells from the toxicity of drugs with a pro-oxidant action such as doxorubicin, cisplatin and EM20-25. The rapid rise in ROS levels prompted by these compounds causes opening of the mitochondrial permeability transition pore (PTP), irreversibly committing cells to death. We find that a fraction of SB3 locates in mitochondrial inner compartments, and that this mitochondrial fraction increases under conditions of oxidative stress. Mitochondrial SB3 inhibits ROS generation and the ensuing PTP induction and cell death through an inhibitory interaction with respiratory Complex I. These findings identify a novel mechanism of action of SB3 that contributes to tumor cell resistance to anti-neoplastic drugs

## INTRODUCTION

Serpins (serine protease inhibitors) are inhibitors of both serine and cysteine proteases [1] characterized by a marked conformational flexibility, which allows to control proteolysis in biological processes as diverse as inflammation, blood coagulation and pressure regulation, chromatin condensation, protein folding, and tumor progression [2, 3]. Serpins can also act independently of their protease inhibitory functions, e.g. as chaperones or hormone transporters [4].

SERPINB3 (SB3), previously known as Squamous Cell Carcinoma Antigen 1 (SCCA1), a member of the ovserpins/clade B serpin family [5], was originally purified from squamous cell carcinoma of the uterine cervix [6].

SB3 is physiologically detected in the superficial and intermediate layers of normal squamous epithelium and it is overexpressed in neoplasms of epithelial or endodermal origins such as lung cancer, head and neck cancer, melanoma, and hepatocellular carcinoma [6-10]. SB3 can neutralize proteinases of the cathepsin family [11], and in cancer cells it confers resistance to drug-induced apoptosis by inhibiting lysosomal cathepsin proteases [12]. However, under a variety of stress conditions SB3 displays an anti-apoptotic function unrelated to its proteinase inhibition activity [6, 13]. Indeed, SB3 protects cells from exposure to radiation through an inhibitory effect either on the MAP family kinase JNK [13] or on p38 [14]; in epithelial ovarian cancer cells exposed to cisplatin, SB3 expression is associated with drug resistance and poor progression-free survival [15], whereas it inhibits the release of mitochondrial cytochrome *c* in squamous cell carcinoma after treatment with TNF-α [16, 17] or with DNA alkylating agents [18]. Moreover, SB3 expression is associated with poor survival in patients with breast cancer treated with anthracycline-based neoadjuvant chemotherapy [19] and in patients with epithelial ovarian cancer a high SB3 expression is a prognostic factor for platinum resistance and shorter progression-free survival [15]. Taken together, these observations suggest that SB3 could favor tumor cell survival under stress conditions, even if the precise molecular mechanisms remain poorly understood.

Most stress and survival signals converge on mitochondria; these organelles are key players in cell death regulation [20] and contribute in several ways to the capability of escaping the lethal effects of stress stimuli that hallmark neoplasms [21]. A key component of the mitochondrial machinery that governs cell death is the permeability transition pore (PTP), an inner membrane channel whose stable opening constitutes a point of no return in cell commitment to death, as it induces mitochondrial depolarization and swelling with massive release of Ca^2+^, and rupture of the outer membrane with release of apoptogenic proteins. [22] A reduced sensitivity of mitochondrial PTP to diverse stress stimuli was described in *in vitro* and *in vivo* models of neoplastic transformation [23, 24], implying that inhibition of pore opening might be a strategy used by tumor cells to avoid death. PTP can be induced by oxidative stress [23, 25], and neoplasms are endowed with an enhanced generation of reactive oxygen species (ROS) compared with non-tumor cells. This altered homeostatic redox equilibrium is caused by several factors, one of the most important being dysregulation of mitochondrial respiratory chain complexes [26], which are the main sites of ROS production in the cell [27]. Thus, in order to set a novel homeostatic redox equilibrium, cancer cells must boost anti-oxidant defenses, and any further increase in ROS levels could overwhelm their residual anti-oxidant capabilities, resulting in the unlocking of PTP desensitization and in the selective killing of malignant cells.

Here we show an unprecedented mitochondrial localization of SB3, which binds respiratory Complex I, down-modulating its activity both in basal conditions and after cell treatment with pro-oxidant chemotherapeutics. By blocking ROS generation at Complex I, SB3 abrogates PTP opening and cell death induced by these drugs, shielding tumor cells from death.

## RESULTS

### SB3 protects from cell death induced by antineoplastic agents

**Figure 1:**
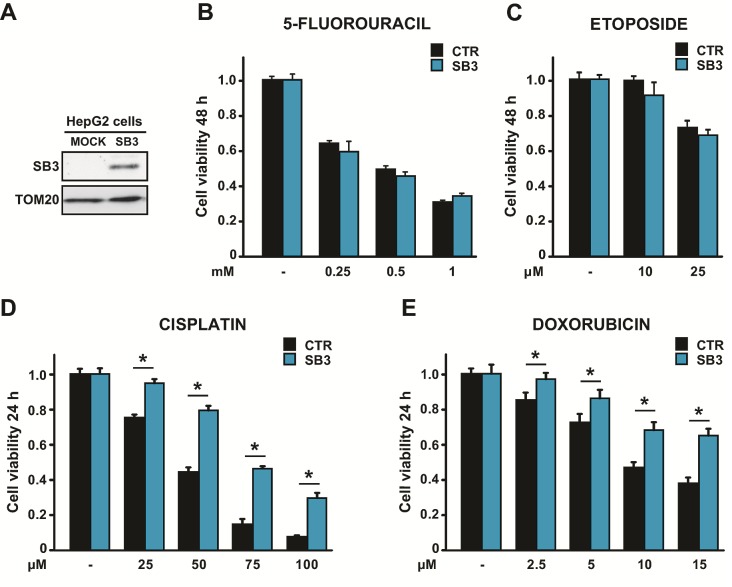
Effect of SB3 expression on the response of HepG2 cells to chemotherapeutics (A) SB3 expression in human hepatoma HepG2 cells stably transfected with a mock construct or with a SB3 plasmid. TOM20 was used as a loading control of the Western immunoblot. (B-E) MTT analysis of cell viability after treatment with the reported concentrations of the chemotherapeutics 5-fluorouracil (B), etoposide (C), cisplatin (D) and doxorubicin (E). Cells were treated for 48 hours with 5-fluorouracil and etoposide, and for 24 hours with cisplatin and doxorubicin. Bars are mean values ±S.D. of tetrazolium salt absorbance for 2×10^4^ recorded cells (n=6, *, p<0.005 with a Student's *t* test).

In cancer cells, SB3 was reported to have an anti-apoptotic activity under a variety of stress conditions [13, 14, 16, 17]. Thus, SB3 could contribute to the ability of tumor cells to escape death. We chose human hepatoma HepG2 and HUH7 cells, which do not show detectable levels of endogenous SB3, as recipient cells to perform a stable SB3 transfection (Fig. 1A and Fig. S1A). To assess the survival role of SB3, we treated cells with a panel of chemotherapeutics: cisplatin, doxorubicin, 5-fluoro-uracil, etoposide and actinomycin D. We found that SB3 expression protected hepatoma cells from the toxicity of both cisplatin and doxorubicin in a dose-dependent fashion, while it was not effective on cells treated with 5-fluoro-uracil, etoposide or actinomycin D (Fig. 1B-E and Fig. S1B-C-F).

### SB3 prevents oxidative stress-induced cell death

**Figure 2:**
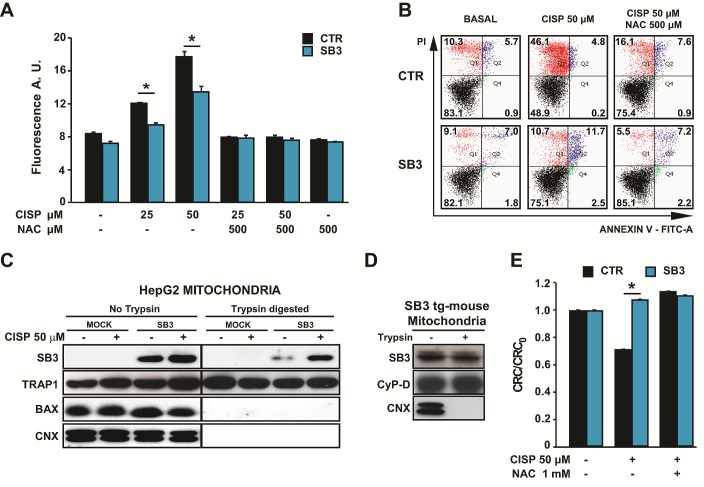
SB3 is located in mitochondria and inhibits oxidative stress and PTP opening (A) Fluorimetric analysis of ROS levels in HepG2 cells treated for 5 hours with the reported cisplatin concentrations with or without N-acetyl-cysteine (NAC). Bars are mean values ±S.D. of CM-H2DCFDA fluorescence (arbitrary units) for 2×10^4^ recorded cells (n=6, *, p<0.005 with a Student's *t* test). (B) Representative traces of cytofluorimetric cell death analysis by Annexin-V and propidium iodide (PI) staining. In black, double negative, viable cells; in green, Annexin-V positive, apoptotic cells; in blue, Annexin-V/PI double positive, late apoptotic cells; in red, PI positive, necrotic cells. Numbers indicate the percentages of cells in each condition. Cells were treated for 24 hours with 50 μM cisplatin; where indicated, NAC was added 1 hour before starting chemotherapeutic treatment. (C) Subcellular fractionation, partial trypsin digestion and Western immunoblot of the mitochondrial fractions of HepG2 cells. TRAP1 was used as a marker of mitochondrial matrix; Bax as a marker of outer mitochondrial membrane; calnexin (CNX) was used as an endoplasmic reticulum marker to check for purity of mitochondrial fractions. 50 μg of mitochondrial proteins were loaded per lane. (D) Partial trypsin digestion and Western immunoblot of wild-type and SB3-transgenic mouse liver mitochondria. Cyclophilin D (CyP-D) was used as a marker of mitochondrial matrix, CNX was used as an endoplasmic reticulum marker to check for purity of mitochondrial fractions. 50 μg of mitochondrial proteins were loaded per lane. (E) PTP opening of HepG2 cells treated with cisplatin is measured with the whole-cell CRC assay. Where indicated, cells were pretreated with N-acetyl-cysteine (NAC). Experiments were performed after 16 hours of drug treatment, *i.e*. before the beginning of the cell death process, and were carried out in a glutamate/malate buffer to maximize Complex I activity. Bars indicate the ratio between the CRC detected in the different experimental conditions (CRC) and in untreated cells (CRC_0_; n=6, *, p<0.005 with a Student's *t* test). A CRC/CRC_0_ lower than 1 indicates PTP induction.

Both cisplatin and doxorubicin elicit a rapid surge of ROS, mainly from mitochondria, independently of their effect as DNA damaging agents [28-31]. We therefore hypothesized that SB3 could prevent cell death caused by oxidative stress. Indeed, SB3 expression strongly inhibited the increase in intracellular ROS levels prompted by cisplatin (Fig. 2A) and doxorubicin (Fig. S1D). Moreover, treatment of hepatoma cells with the anti-oxidant compound N-acetyl-cysteine (NAC) mimicked the effect of SB3, in that it markedly inhibited both the ROS surge (Fig. 2A and Fig. S1D) and death induction caused by cisplatin and doxorubicin (Fig. 2B and Fig. S1B and S1E). Similar to SB3 expression, NAC could not protect hepatoma cells from toxicity elicited by 5-fluoro-uracil, etoposide or actinomycin D (Fig. S1C and S1F), which indicates that these drugs do not induce oxidative stress, and further support the hypothesis of an anti-oxidant role of SB3.

### Mitochondrial localization of SB3

One of the main sites of ROS production in the cell is the mitochondrial respiratory chain, and mitochondria contribute to the neoplastic process by regulating redox equilibrium changes [27], energy metabolism [32] and cell death [20]. We therefore evaluated the possibility that SB3 could regulate ROS levels by directly interacting with some mitochondrial components. In keeping with this hypothesis, we found that a significant proportion of SB3 was protected from digestion by trypsin concentrations able to degrade proteins of the outer mitochondrial membrane (Fig. 2C and Fig. S1G), indicating that SB3 mainly locates in the inner mitochondrial compartments; and that treatment with cisplatin increased the mitochondrial fraction of SB3 (Fig. 2C). As a mitochondrial localization of SB3 was never reported before, we checked for its subcellular distribution in transgenic mice expressing SB3 in the liver [33] and in other human cancer cell models, including H295R adrenocortical carcinoma cells and SAOS-2 osteosarcoma cells, confirming that SB3 localizes in the mitochondrial matrix (Fig. 2D and Fig. S1G-H).

### SB3 abrogates PTP opening induced by oxidative stress

Taken together, these observations suggest that SB3 displays a protective function against oxidative insults by impinging upon mitochondrial redox equilibrium. Increased levels of mitochondrial ROS can lead to cell death through opening of the PTP [23]. To test if SB3 shields cells from oxidative stress by inhibiting ROS-dependent pore opening we performed a whole-cell Ca^2+^-retention capacity (CRC) assay [34], which measures the Ca^2+^ threshold for PTP opening in permeabilized cells exposed to a train of repeated Ca^2+^ pulses, as accumulation of Ca^2+^ into mitochondria induces the PTP. Since Complex I is one of the main sites of superoxide production in the cell [35], this experiment was carried out under conditions that maximize the activity of respiratory chain Complex I, *i.e*. in a buffer containing glutamate/malate. Our data indicate that in HepG2 cells cisplatin sensitizes the PTP to Ca^2+^ in a ROS-dependent way, as its effect is inhibited by NAC; and, remarkably, that SB3 expression abrogates PTP induction by cisplatin (Fig. 2E).

**Figure 3:**
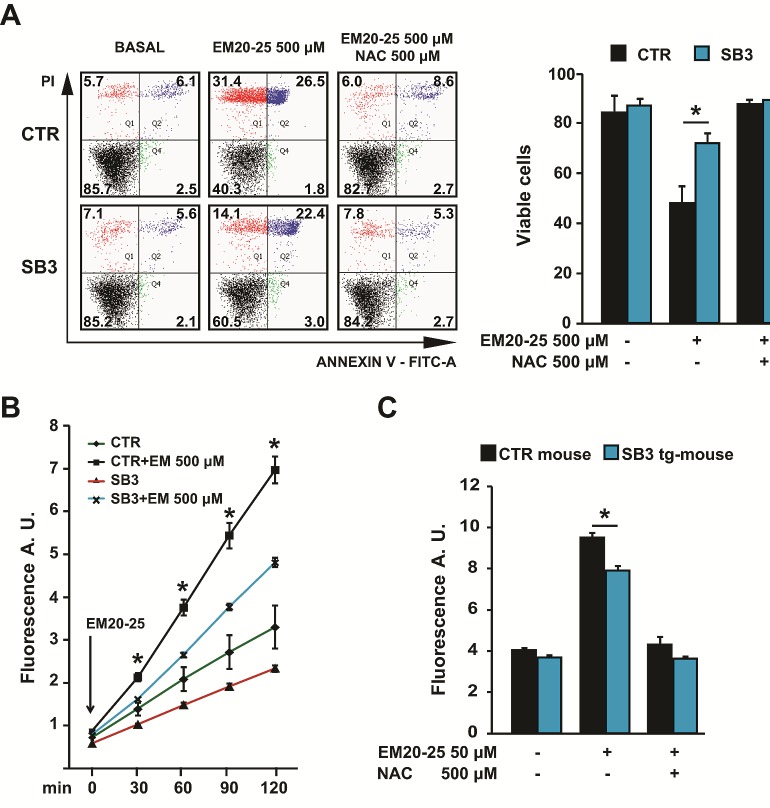
SB3 protects from oxidative stress and cell death elicited by the PTP opener EM20-25 (A) Representative traces of cytofluorimetric cell death analysis by Annexin-V and propidium iodide (PI) staining. In black, double negative, viable cells; in green, Annexin-V positive, apoptotic cells; in blue, Annexin-V/PI double positive, late apoptotic cells; in red, PI positive, necrotic cells. Numbers indicate the percentages of cells in each condition. Cells were treated for 2h30min with EM 500 μM; where indicated, 500 μM N-acetyl-cysteine (NAC) was added 1 hour before starting chemotherapeutic treatment. (B, C) Fluorimetric analysis of ROS levels in HepG2 cells (B) and in wild-type and SB3-transgenic mouse liver mitochondria (C) treated for 2 hours with the reported EM20-25 concentrations; where indicated, N-acetyl-cysteine (NAC) was added 1 hour before starting the experiment. Bars are mean values ±S.D. of CM-H2DCFDA fluorescence (arbitrary units) for 2×10^4^ recorded cells or 200 μg mitochondria (n=6, *, p<0.005 with a Student's *t* test).

To gain further insights on the mechanisms by which SB3 protects from PTP opening, we looked for additional compounds that induce oxidative stress and the ensuing PTP opening. The BH3 mimetic EM20-25, a well-characterized PTP opener in several tumor cell types [36], elicited a massive and fast death in HepG2 mock cells, which was paralleled by a rapid increase in ROS levels (Fig. 3A and 3B). When cells were pre-treated with NAC, EM20-25 toxicity was totally abolished (Fig. 3A), indicating the major involvement of oxidative stress in the observed EM20-25 effect. Notably, SB3 expression shielded HepG2 cells from both EM20-25 lethality and from the ROS surge elicited by the drug (Fig. 3A and 3B). EM20-25 displayed a clear pro-oxidant effect also in mouse liver mitochondria, where significant protection by SB3 was confirmed as well (Fig. 3C).

### SB3 interaction with Complex I

**Figure 4:**
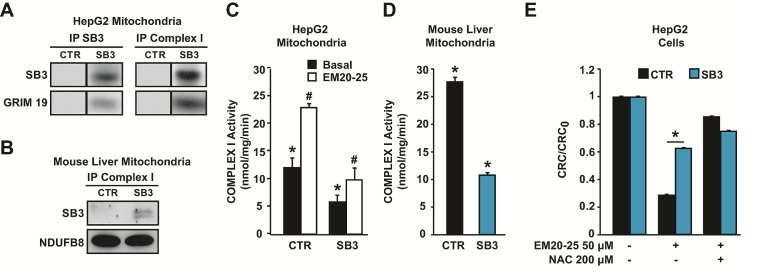
SB3 interacts with respiratory Complex I and inhibits its enzymatic activity (A, B) SB3 and Complex I and TRAP1 immunoprecipitations (IPs) on mitochondria from HepG2 cells (A) and on wild-type and SB3-transgenic mouse liver mitochondria (B). The interaction between SB3 and Complex I is shown by co-IP; to detect Complex I, two different subunits (GRIM19 and NDUFB8) were probed. (C, D) Spectrophotometric analysis of the NADH:ubiquinone oxidoreductase enzymatic activity of complex I in mitochondria from HepG2 cells (C) or from mouse liver (D); both in (C) and in (D), n=6, *, p<0.005 with a Student's t test). (E) PTP opening of HepG2 cells treated with EM20-25 is measured with the whole-cell CRC assay. Where indicated, cells were pretreated with N-acetyl-cysteine (NAC). Experiments were carried out in a glutamate/malate buffer to maximize Complex I activity. Bars indicate the ratio between the CRC detected in the different experimental conditions (CRC) and in untreated cells (CRC0; n=6, *, p<0.005 with a Student's t test). A CRC/CRC0 lower than 1 indicates PTP induction.

High levels of ROS can be generated in mitochondria by compounds that modulate the activity of respiratory complexes on the inner membrane. As we found SB3 in inner mitochondrial compartments, we investigated if it could directly interact with the respiratory chain. Co-immunoprecipitations experiments revealed an association between respiratory Complex I and SB3 both in HepG2-SB3 cells and in liver mitochondria of SB3 transgenic mice (Fig. 4A and 4B). Importantly, the physical association between SB3 and Complex I had a functional effect, as the presence of SB3 markedly inhibited the enzymatic activity of Complex I of both cultured cells and mouse liver mitochondria (Fig. 4C and 4D).

Dysregulation of ROS generated by Complex I could trigger an oxidative stress capable of opening the PTP [37]. Indeed, the PTP opener EM20-25 strongly increased Complex I activity, and SB3 totally blunted Complex I induction by EM20-25 (Fig. 4C). Thus, modulation of Complex I activity could have a key role on redox equilibrium, and on PTP opening elicited by oxidative insults. Accordingly, CRC experiments carried out in a glutamate/malate buffer demonstrated that EM20-25 induces the PTP in a ROS-dependent way, as this induction was abrogated by NAC-dependent ROS scavenging (Fig. 4E). Remarkably, SB3 expression had an inhibitory effect on PTP induction that closely resembled that of NAC (Fig. 4E). Taken together, these observations indicate that SB3 protects cells from the lethal effects of oxidative insults by inhibiting ROS generation by Complex I and the ensuing PTP opening.

## DISCUSSION

In the present work we have characterized a novel activity of SB3 that may have important implications in the tumorigenic process. We have found that a fraction of SB3 is located in the inner mitochondrial compartments, where it binds respiratory Complex I and down-modulates its activity. By a parallel inhibition of ROS generation at Complex I, SB3 shields cells from the noxious effects of oxidative stress, in particular from death-inducing PTP opening caused by chemotherapeutic agents that also act as pro-oxidants. Since SB3 is over-expressed in several epithelial tumors [6, 7, 9] and in preneoplastic liver lesions, [8] it can be considered an important component of the anti-apoptotic machinery installed by neoplastic cells to survive the variety of stress conditions they encounter during malignant progression. In addition, the recently described resistance to chemotherapeutics and the poor prognosis of patients with both breast [19] and ovarian cancer [15] over-expressing SB3 are in keeping with the results of this study.

Beside the antiprotease activity, a high degree of functional pleiotropy has been associated with SB3, including involvement in tissue remodeling during fibrosis associated to autoimmune diseases [6, 38], induction of TGF-β [39, 40] and of the oncogene Myc [41] and hepatocyte proliferation after partial hepatectomy [33]. Together with its anti-apoptotic activity, all these functions of SB3 could contribute to neoplastic progression. Indeed, inhibition of NK cell recruitment, and of NK cell-induced apoptosis [1], could attenuate the anti-tumor innate immunity response, which is consistent with increased tumor infiltration by NK cells in *in vivo* cancer models after SB3 inhibition [17]. Furthermore, SB3 expression was shown to prompt tissue remodeling and cell proliferation associated with increased β-catenin accumulation, which could play a role in epithelial mesenchymal transition [42] and invasion [43] of tumor cells.

Notably, the SB3-mediated modulation of redox that we report here could be part of this complex picture, as ROS contribute in stimulating proliferation, invasion and metastasis, and in inhibiting apoptosis [44]. Indeed, ROS play complex and multiple roles in the tumorigenic process. Several oncogenes increase ROS levels by unbalancing the energy metabolism pathways of the tumor cell, which can cause electron leakage during oxidative phosphorylation and stimulate generation of superoxide; and a surge in mitochondrial ROS stimulates cellular proliferation and anchorage-independent growth [45]. Nonetheless, tumor cells must reach a novel redox equilibrium, to avoid an uncontrolled rise in ROS levels that would trigger a feed-forward loop involving a progressive rise in Ca^2+^ concentration, a further ROS increase and eventually PTP opening and death [23, 25]. Neoplasms must therefore utilize several strategies to maintain a locked PTP, *e.g*. by the association of hexokinase II with mitochondria [34], or by signalling conveyed by kinases [24], such as a mitochondrial branch of an ERK/GSK-3β signalling axis [36]. In this framework, tumor cells could exploit SB3 as a further mechanism of defense from oxidative insults.

The diverse functions of SB3 are paralleled by its multiple locations, as the protein can be found both in nuclei and cytoplasm, and it can also be released from tumor cells [3], acting through both autocrine and paracrine pathways [42] and serving as tumoral or prognostic marker [46]. Here we have identified a novel site of SB3 expression in the mitochondrial matrix, where it is involved in an unprecedented function, *i.e*. protection from oxidative stress through interaction with the respiratory Complex I. Notably, SB3 further translocates into mitochondria following cisplatin treatment, which suggests its recruitment as a component of the mitochondrial anti-oxidant machinery. Most of the ROS generated in intact mitochondria are contributed by the NADH:quinone oxidoreductase activity of this enzyme [35]. The importance of Complex I-dependent oxidants is further highlighted by our recent observation that the Gold(III)-dithiocarbamato compound AUL12 induces neoplastic cell death by enhancing ROS generation at Complex I, thus triggering multiple signalling pathways that eventually lead to PTP opening [37]. The importance of regulation of respiratory chain complexes in cancer cells is becoming increasingly clear, as exemplified by the recent observations that activity of Complex I is inhibited in models of K-Ras-driven tumorigenesis [47], and that down-regulation of Complex II by the mitochondrial chaperone TRAP1 creates a pseudohypoxic phenotype that promotes tumorigenesis [48].

Redox alterations are postulated to make neoplastic cells particularly vulnerable to oxidants, and indeed ROS generation contributes to the toxicity of a variety of chemotherapeutics. Therefore, increased understanding of the molecular mechanisms by which SB3 protects tumor cells from oxidants has possible implications for developing anti-neoplastic strategies that target the altered redox equilibrium of SB3-expressing neoplasms.

## METHODS

### Ethics statement

Experiments with mice were authorized by the Animal Care and Use Committee of the University of Padova and carried out according to European guidelines.

### Cell culture transfections and SB3-transgenic mice

HepG2 cells were cultured in Minimum Essential Medium (MEM; Sigma, St. Louis, Missouri) added with 10% Fetal Bovine Serum (FBS; Invitrogen Life Technologies, Carlsbad - California), glutamine 2 mM (Sigma), MEM non essential amino acids (Sigma), penicillin (100 units/ml), streptomycin (100 μg/ml; Invitrogen), and were kept in a humidified atmosphere of 5% CO2 at 37°C. Cells were stably transfected with a vector carrying the full length human SB3 cDNA inserted in a pCDNA3.1 vector, mock cells were obtained by stable transfection with an empty pCDNA3.1 plasmid, as described [42]. The study was carried out in C57BL/6 mice transgenic for human SB3 [33]. Mice were bred at the Animal Care Facility of the Experimental Surgery Division of the University of Padua.

### Cell viability assays

To analyze viability after chemotherapeutic treatments, HepG2 cells were plated in 96-well plate (2×10^4^ cells/well) and synchronized by a 24 hour FBS depletion. FBS was then re-added in the medium at the time of chemotherapeutic addition. Cell viability was assessed either with a MTS assay or by flow cytometry analysis. The MTS assay (CellTiter 96® AQueous One Solution; Promega, Madison, Wisconsin) exploits an improved tetrazolium compound, MTS (3-(4,5-dimethylthiazol-2-yl)-5-(3-carboxymethoxyphenyl)-2-(4-sulfophenyl)-2H-tetrazolium, inner salt), that is reduced by dehydrogenase enzymes of living cells into a formazan product whose absorbance at 490 nm is directly proportional to the number of viable cells. To perform colorimetric measurements, cells were washed with PBS and then a HBSS solution containing MTS was added. Plates were incubated at 37°C for 1 h and read in a Multiskan EX multiplate reader (Thermo Scientific,, Waltham, Massachusetts). Flow cytometry recordings were performed as described previously [49, 50] to detect phosphatidylserine exposure on the cell surface (increased FITC-conjugated Annexin-V staining) and loss of plasma membrane integrity (propidium iodide staining, 1 μg/ml). Samples were analyzed on a FACSCanto II flow cytometer (Becton Dickinson, Franklin Lakes, New Jersey). Data acquisition and analysis were performed using FACSDiva software (Becton Dickinson).

### Preparation of mitochondrial extracts

To prepare mitochondrial extracts, either cells or minced mouse livers were placed in an isolation buffer (250 mM sucrose, 10 mM Tris/HCl, 10 mM EGTA/Tris, pH 7.4 with phosphatase and protease inhibitors) and homogenized at 4°C with an electrical glass-Teflon potter. Mitochondria were then isolated by differential centrifugation (three times, the first at 700 × *g* to eliminate nuclei, plasma membrane fractions and undisrupted cells and twice at 7,000 × *g*, all at 4°C, 10 minutes each to pellet and wash mitochondria) in mitochondrial isolation buffer. Protease digestion of isolated mitochondria was performed in isolation buffer without protease inhibitors for 1 h at 4°C. After inactivating trypsin with a protease inhibitor cocktail (Sigma), mitochondria were spun (18,000 × *g* for 10 minutes) and lysed.

### Western blot analyses and immunoprecipitations

Total cell extracts were prepared at 4°C in 140 mM NaCl, 20 mM Tris/HCl (pH 7.4), 5mM EDTA, 10% glycerol, and 1% Triton ×-100 in the presence of phosphatase and protease inhibitors (Sigma). All lysates were kept for 30 minutes on ice and then cleared by centrifugation at 4°C and 14,000 × *g* for 25 minutes. Protein quantification was performed with BCA Protein Assay Kit (Thermo Scientific-Pierce).

Immunoprecipitations were performed on 1 mg of proteins extracted from total cell lysates and on 300 μg from isolated mitochondria cleared with protein A-Sepharose (Sigma) for 1 h at 4°C. Incubation with the primary antibody (1 μg per sample) was carried out in agitation at 4°C for 16 hours. Western immunoblots were carried out under standard conditions, and proteins were transferred onto nitrocellulose Hybond-C Extra membranes (Amersham, Uppsala - Sweden) and visualized by enhanced chemiluminescence (Millipore, Milan, Italy).

Anti-SERPINB3 rabbit polyclonal antibody was from Xeptagen (Venezia, Italy); mouse monoclonal anti-SERPINB3 (sc-21767), anti Grim19 (sc-271013) and anti TRAP1 (sc-13557) antibodies, rabbit polyclonal anti TOM20 (sc-11415), anti Bax (sc-493) and anti Calnexin (sc-6465) antibodies were all from Santa Cruz (Santa Cruz, California); mouse monoclonal anti CyP-D antibody (AP1035) was from Calbiochem (Darmstadt, Germany); mouse monoclonal anti GAPDH (MAB374) was from Merck Millipore (Billerica, Massachusetts); mouse monoclonal anti NDUFB8 (20E9DH10C12) and mouse monoclonal anti Complex I Immunocapture kit (ab109711) antibodies were from MitoSciences (Eugene, Oregon).

### ROS measurements

To perform ROS measurements, cells were plated as for cell viability assays; isolated mitochondria (200 μg/well) were resuspended in a buffer containing 250 mM Sucrose, 10 mM MOPS-Tris, 5 mM Glutamate, 2.5 mM Malate, 4 mM MgCl_2_, 1 mM Pi-Tris, at pH 7.4. ROS levels were detected by using the probe 5-(and-6)-chloromethyl-2’,7’-dichlorodihydrofluorescein diacetate, acetyl ester (CM-H2DCFDA, 1 μM; Molecular Probes, Carlsbad, - California). Fluorescence (λ exc: 485 nm; λ em: 538 nm) was recorded using a Fluoroskan Ascent FL (Thermo Electron Corp.) plate reader.

### Respiratory Complex I activity assay and Citrate Synthase activity assay

The enzymatic activity of respiratory chain Complex I was assessed on freshly isolated mitochondria or permeabilized cells (40 μg of proteins per trace). Samples were pre-incubated for 3 min at 37°C in a buffer composed by potassium phosphate 25 mM pH 7.4, alamethicin 1 μM, BSA 3 mg/ml, sodium azide (500 μM), 6.5 μM Coenzyme Q1. Reaction started after the addition of NADH 10 μM and NADH consumption was recorded spectrophotometrically as a decrease in absorbance at a 340 nm wavelength for 5 minutes. The Complex I activity was calculated as NADH consumption assessing the difference of the trace slopes with or without the Complex I inhibitor Rotenone 10 μM. Each measurement of Complex I activity was normalized for Citrate Synthase (CS) activity. Citrate formation was spectrophotometrically measured as an increase in absorbance at 420 nm wavelength in a buffer containing 100 mM Tris-HCl pH 8, 100 μM DTNB, 300 μM Acetyl -CoA, 500 μM Oxaloacetate, at 37°C.

### Measurement of Mitochondrial Ca^2+^ Retention Capacity

The CRC assay was performed as described to assess PTP opening in whole cells exposed to trains of Ca^2+^ pulses [34, 36]. Briefly, cells were permeabilized with 100 μM digitonin (15 min, 4°C) in a high (1 mM) EGTA buffer. Digitonin was then eliminated and permeabilized cells were placed in low (10 μM) EGTA in the presence of 5 mM Glutamate/2.5 mM Malate, 10 μM cytochrome *c*, and of the Ca^2+^ probe Calcium Green-5N (1 μM; λ exc: 505 nm; λ em: 535 nm; Molecular Probes), which does not permeate mitochondria. Cells were then exposed to Ca^2+^ spikes, and fluorescence drops were used to assess mitochondrial Ca^2+^ uptake using a Fluoroskan Ascent FL (Thermo Electron Corp.) plate reader. PTP opening was detected as a sudden and irreversible fluorescence increase. The Ca^2+^ taken up by mitochondria before PTP opening in the different experimental conditions was then normalized to control conditions (dubbed as CRC_0_).

## SUPPLEMENTARY FIGURE


