# Supplementary material for: SERPINB3 protects from oxidative damage by chemotherapeutics through inhibition of mitochondrial respiratory complex I
Source: Oncotarget. 2013 Dec 24;5(9):2418–27. doi: 10.18632/oncotarget.1411 (PMC4058015; doi:10.18632/oncotarget.1411)
Supplement: Supplementary file 1 [file oncotarget-05-2418-s001.pdf]

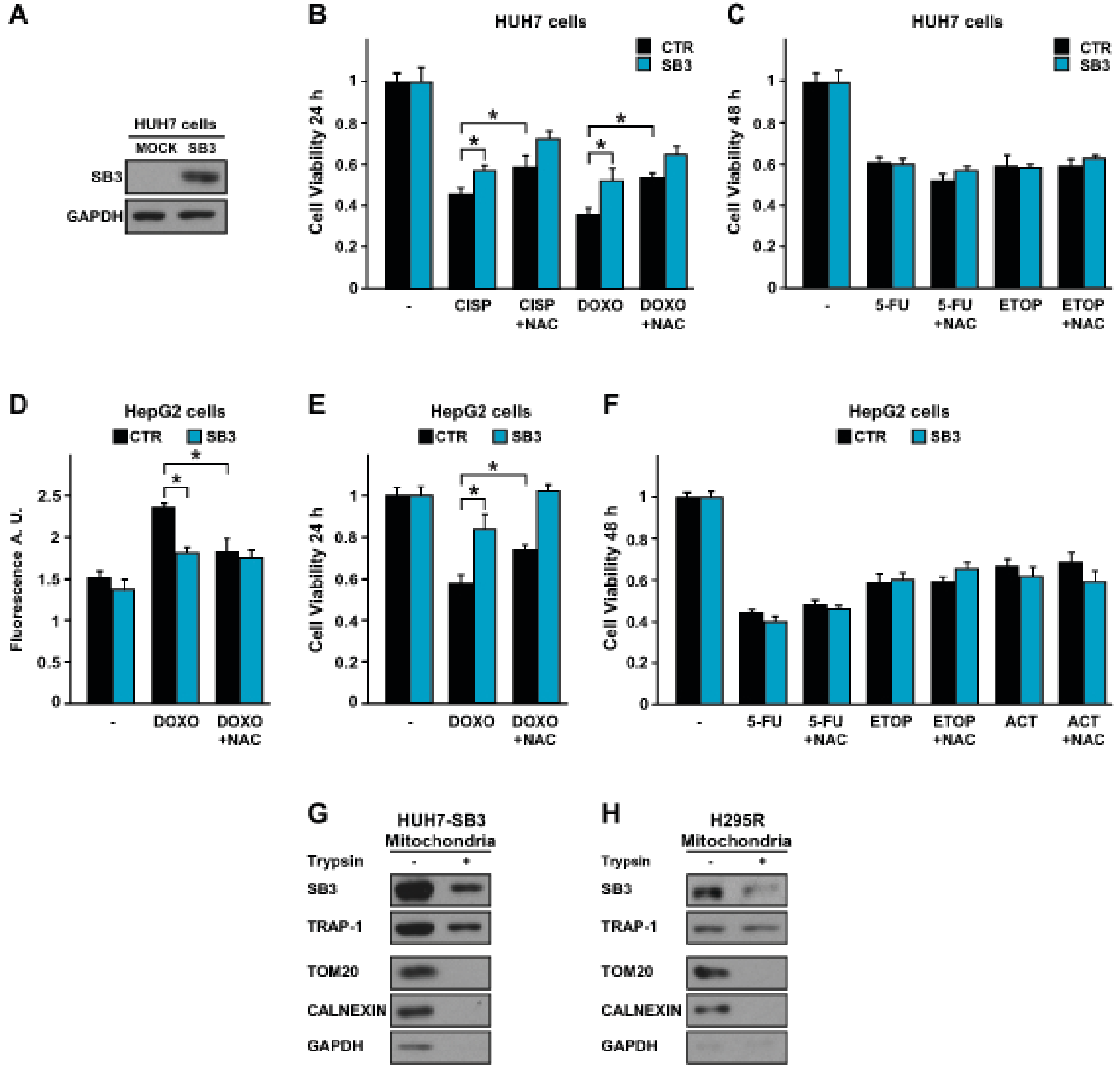

Supplementary Figure 1: Effect of SB3 expression on the response of HepG2 and HUH7 cells to chemotherapeutics. (A) SB3 expression in human hepatoma HUH7 cells stably transfected with a mock construct or with a SB3 plasmid. GAPDH was used as a loading control of the Western immunoblot. (B, C) MTT analysis of HUH7 cell viability after treatment with cisplatin (50 µM, 24 hours), doxorubicin (5 µg/ml, 24 hours), 5-fluorouracil (1 mM, 48 hours), etoposide (50 µM, 48 hours) Bars are mean values ±S.D. of tetrazolium salt absorbance for $1x10^4$ recorded cells (n=6, *, $p<0.005$ with a Student's t test). (D) Fluorimetric analysis of ROS levels in HepG2 cells treated with doxorubicin (10µg/ml, 1 hour). Bars are mean values ±S.D. of CM-H2DCFDA fluorescence (arbitrary units) for $2x10^4$ recorded cells (n=4, *, $p<0.05$ with a Student's t test). (E, F) MTT analysis of HepG2 cell viability after treatment with doxorubicin (5 µg/ml, 24 hours), 5-fluorouracil (1 mM, 48 hours), etoposide (50 µM, 48 hours). Data are shown as in (B, C). (G, H) Partial trypsin digestion followed by Western immunoblot of the mitochondrial fractions of HUH7 cells (G) and H295R cells (H). TRAP1 was used as a marker of mitochondrial matrix; TOM20 as a marker of outer mitochondrial membrane; calnexin and GAPDH were used to check for purity of mitochondrial fractions; 40 µg of mitochondrial proteins were loaded per lane.
All along the Figure, 500 µM N-acetyl-cysteine (NAC) was added 1 hour before starting chemotherapeutic treatment.
